# Understanding the intrinsic radioactivity energy spectrum from ^176^Lu in LYSO/LSO scintillation crystals

**DOI:** 10.1038/s41598-018-35684-x

**Published:** 2018-11-23

**Authors:** H. Alva-Sánchez, A. Zepeda-Barrios, V. D. Díaz-Martínez, T. Murrieta-Rodríguez, A. Martínez-Dávalos, M. Rodríguez-Villafuerte

**Affiliations:** 1 Instituto de Física, Universidad Nacional Autónoma de México, A.P. 20–364, 01000 Mexico City, Mexico; 2 Facultad de Ciencias, Universidad Nacional Autónoma de México, 04510 Mexico City, Mexico

## Abstract

Lutetium oxyorthosilicate (LSO) or lutetium yttrium oxyorthosilicate (LYSO) are the scintillator materials most widely used today in PET detectors due to their convenient physical properties for the detection of 511 keV annihilation photons. Natural lutetium contains 2.6% of ^176^Lu which decays beta to excited states of ^176^Hf producing a constant background signal. Although previous works have studied the background activity from LSO/LYSO, the shape of the spectrum, resulting from β-particle and γ radiation self-detection, has not been fully explained. The present work examines the contribution of the different β-particle and γ-ray interactions to provide a fuller comprehension of this background spectrum and to explain the differences observed when using crystals of different sizes. To this purpose we have shifted the continuous β-particle energy spectrum of ^176^Lu from zero to the corresponding energy value for all combinations of the isomeric transitions of ^176^Hf (γ-rays/internal conversion). The area of each shifted β-spectrum was normalized to reflect the probability of occurrence. To account for the probability of the γ-rays escaping from the crystal, Monte Carlo simulations using PENELOPE were performed in which point-like sources of monoenergetic photons were generated, inside LYSO square base prisms (all 1 cm thick) of different sizes: 1.0 cm to 5.74 cm. The analytic distributions were convolved using a varying Gaussian function to account for the measured energy resolution. The calculated spectra were compared to those obtained experimentally using monolithic crystals of the same dimensions coupled to SiPM arrays. Our results are in very good agreement with the experiment, and even explain the differences observed due to crystal size. This work may prove useful to calibrate and assess detector performance, and to measure energy resolution at different energy values.

## Introduction

To date, most clinical and preclinical positron emission imaging systems, combine scintillation crystals coupled to position-sensitive photodetectors like photomultiplier tubes or silicon photomultipliers (SiPM). Cerium doped lutetium oxyorthosilicate Lu_2_SiO_5_:Ce (LSO) or lutetium yttrium oxyorthosilicate, Lu_2(1-*x*)_Y_2*x*_SiO_5_:Ce (LYSO), originally discovered by C.L. Melcher and J.S. Schweitzer^1^, are the scintillator materials most widely used today in PET detectors^2,3^ due to their convenient physical properties for the detection of 511 keV annihilation photons, including high light output, high linear attenuation coefficient and short decay time^4^. Other inorganic scintillators containing lutetium, such as LuAlO_3_:Ce (LuAP) and Lu_2_Si_2_O_7_:Ce (LPS) or Lu_2_O_3_:Eu, have been also investigated as candidates for nuclear medicine detectors^3–5^. Natural lutetium contains about 2.6% of ^176^Lu which decays by beta emission with mean and maximum β-particle energy of 182 keV and 593 keV, respectively, to excited states of ^176^Hf with a half-life of 3.76 × 10^10^ years producing a constant background signal, which can be removed by means of coincidence detection. However, this intrinsic radioactivity may have an impact when imaging low levels of activity, especially when using wide energy windows^6,7^ and even more when designing detectors for single photon imaging when background emissions tally with those used in SPECT scanners that have LSO/LYSO scintillators^8^. On the other hand, this background signal can be conveniently used to produce flood-source images and energy spectra to verify detector functioning in singles mode^9,10^.

Previous works have studied the background activity from LSO/LYSO for positron emission imaging applications. Yamamoto, S. *et al*.^11^, for example, observed the typical broad spectrum from an LSO crystal with three main peaks and suggested that these probably resulted from the simultaneous detection of the β-particle plus 88 keV γ-ray, β-particle plus 202 keV γ-ray and β-particle plus 307 keV γ-ray, respectively, but did not deepen into the relative intensities of the peaks. This was also pointed out in other works for LYSO crystals^12,13^. More recently, Jeong, M. *et al*.^14^ published the energy spectrum from two pixelated LYSO crystal sizes (50.8 × 50.8 × 5 mm^3^ and 50.8 × 50.8 × 10 mm^3^), but did not dwell upon the clearly visible differences in the relative intensities of the broad peaks.

As far as we know, the shape of the spectrum resulting from β-particle and γ-radiation self-detection, and its dependence on the size of the scintillation crystal, has not been fully explained. The present work aims at providing a more detailed explanation of the structure of the LSO/LYSO scintillation intrinsic radioactivity energy spectrum due to the β and γ radiation from ^176^Lu present in natural lutetium and to explain the differences observed when using crystals of different dimensions.

## Methods

### Calculations

**Figure 1:**
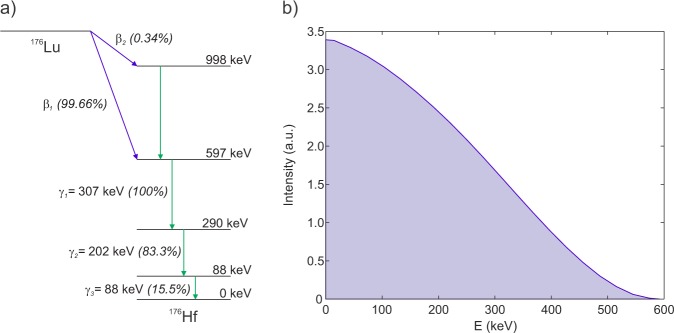
(**a**) Simplified ^176^Lu decay scheme and (**b**) β-particle energy spectrum corresponding to the β_1_ transition.

^176^Lu decays by beta-emission followed by one or more prompt γ-ray emissions with different associated probabilities^15^ (Fig. 1a). The background energy spectrum is therefore a result of several contributions: a) the energy deposited by β-particles, b) secondary electrons produced by γ-ray interactions and c) conversion electrons from internal conversion (IC) processes, the latter (b and c) arising from the excited states of ^176^Hf. To calculate the contribution of the different combinations of simultaneous β-particle and γ-ray self-detection in the crystal, the continuous β_1_-particle energy spectrum of ^176^Lu, shown in Fig. 1b (data taken from Eckerman, K. F. *et al*.^16^), was shifted from zero to the corresponding energy value (the β_2_ branch with a probability of 0.34% was not considered in the calculations). The area of each shifted β-spectrum was normalized to reflect the probability of occurrence of each combination, calculated from the probability values^15^ of the isomeric transitions of ^176^Hf considering γ-ray emission and the IC process. In the calculation it was assumed that all β-particles and IC electrons deposit all their energy within the crystal, an assumption which was verified using Monte Carlo simulations (see below). In addition, the low-energy X-rays resulting from ionizations in the crystal and Hf vacancies, reported by Norman, E. B. *et al*.^17^ were assumed to deposit all of their energy via photoelectric interactions in the crystal.

To account for the probability of the 88, 202 and 307 keV γ-rays escaping from the crystal, Monte Carlo simulations using PENELOPE^18^ were performed. With this code, point-like sources of monoenergetic photons were generated, evenly distributed inside LYSO crystals of two different dimensions: a 1.0 cm cube and a square base prism of dimensions 5.74 × 5.74 × 1.00 cm^3^. A 10 keV cut-off energy value, below which particles are assumed to be stopped and absorbed in the medium, was used for photons and secondary electrons. From the simulations, the probability of the prompt γ-rays detected within the crystal was obtained (Table 1). Scattered photons escaping the crystal, which account for a small fraction of the total generated photons, were not considered.

**Table 1:** Probabilities of the different decay modes and γ-ray interactions for two LYSO crystal sizes.

ID	γ-ray transition energy (keV)	Probabilities			
		γ-ray emission	Internal conversion (IC)	γ-ray absorbed in the crystal^a^	γ-ray escapes the crystal^a^
Small crystal (1 × 1 × 1 cm^3^)					
γ_1_	307	1	0	0.382	0.618
γ_2_ or IC_2_	202	0.833	0.167	0.654	0.346
γ_3_ or IC_3_	88	0.155	0.845	0.949	0.051
Big crystal (5.74 × 5.74 × 1 cm^3^)					
γ_1_	307	1	0	0.603	0.397
γ_2_ or IC_2_	202	0.833	0.167	0.815	0.185
γ_3_ or IC_3_	88	0.155	0.845	0.977	0.023

^a^Probabilities calculated with PENELOPE as described in the text.

To verify that most of the electrons deposit their energy within the crystal volume, we performed a simulation using electrons as primary particles in the 1 cm cube generated randomly and evenly distributed within the whole crystal volume with an energy spectrum of the β^−^ decay of Lu-176 shown in Fig. 1b (with endpoint energy = 593 keV). In this case a 0.1 keV cut-off energy value was used. The results indicate that 99.3% of the electrons are absorbed in the crystal (0.7% escape from the crystal surface).

Table 2 shows the energy deposition probabilities of each event combination for both crystal sizes. These values consider the combined probability of γ-ray emission and its detection within the crystal together with (or without) internal conversion processes; values are shown for two crystal sizes. For example, for an event in which the β-particle is detected simultaneously with both an 88 keV isomeric transition and a 202 keV isomeric transition, four detection combinations are possible, in all of which the γ_1_-ray escapes from the crystal: (i) γ_2_ + γ_3_, (ii) IC_2_ + γ_3,_ (iii) γ _2_ + IC_3,_ and (iv) IC_2_ + IC_3_. For the 1 cm cube crystal, the probabilities for each combination are 0.0495, 0.0152, 0.2842 and 0.0872, respectively. Thus, the probability that a total energy of 290 keV is deposited (together with the β-particle energy) is the sum of these values, which add up to 0.436. Likewise, for the large square prism, these probabilities are 0.041, 0.010, 0.228 and 0.056, which add up to 0.335 (fourth row in Table 2). The probability that all three γ-rays are emitted and that none are detected in the crystal (i.e. the three of them escape and thus 0 keV is deposited in the crystal due to γ-ray interactions) is very low: 0.0014 and 0.0002 for the small and large crystals, respectively. In this case, the β-particle spectrum is not shifted, but only scaled-down (area normalization) to reflect this rare occurrence.

**Table 2:** Probabilities of the combined events for two crystal sizes.

Energy deposited in the crystal from isomeric transition combinations (keV)	Sum Energy (keV)	Probabilities	
		Small Crystal	Large Crystal
0	0	0.0014	0.0002
88	88	0.1767	0.0610
202	202	0.0035	0.0012
88 + 202	290	0.4360	0.3349
307	307	0.0009	0.0003
88 + 307	395	0.1094	0.0925
202 + 307	509	0.0022	0.0018
88 + 202 + 307	597	0.2700	0.5079
	Sum	1.0000	1.0000

The set of shifted β-particle spectra were summed considering their weight (given by the probability of occurrence) to produce the final expected energy spectrum. To take into consideration the measured energy spectrum of LYSO, the analytic distributions were convolved using a variable Gaussian function obtained experimentally with a standard deviation of the form σ(*E*) = 1.15*E*^0.52^ keV. This function is in good agreement with the theoretical dependence of the energy resolution *R*_E_ with the energy *E*, in which, for Poisson-dominant systems is *R*_E_ ∝ *E*^−1/2^ as described in G.F. Knoll^19^.

### Experiment

Energy spectra from two monolithic LYSO crystals with all surfaces polished (Proteus Inc., Chagrin Falls, OH, USA) having the aforementioned sizes were acquired using a SiPM array (ArrayC-60035-64P by SensL Technologies Ltd. Cork, Ireland) under controlled conditions of ambient light, in a setup described in detail in Calva-Coraza E. *et al*.^20^. No special detector cooling system was employed.

The crystals were wrapped on five sides with white Teflon tape, and the energy spectra were calibrated using sealed γ-ray sources: ^22^Na (511 keV, 1275 keV) and ^137^Cs (662 keV). Acquisitions of the background radiation (no external sources) were performed for 60 min and 15 min for the small cube and large prism; the count rates per cm^3^ of LYSO were 289 cps and 267 cps, respectively. The lower count rate per unit volume for the large prism can be explained by the fact that the small crystal has a larger surface area (covered in white) to volume ratio compared to the large one, and hence more light per unit volume is being reflected to the photodetector in the small cube than in the large prism. In addition, there is more self-absorption of optical photons in the large crystal.

For both crystal sizes the experimental and calculated energy spectra were normalized to have the area under the curve equal to 1.0 and plotted in the same graph for comparison.

## Results

### Calculations

**Figure 2:**
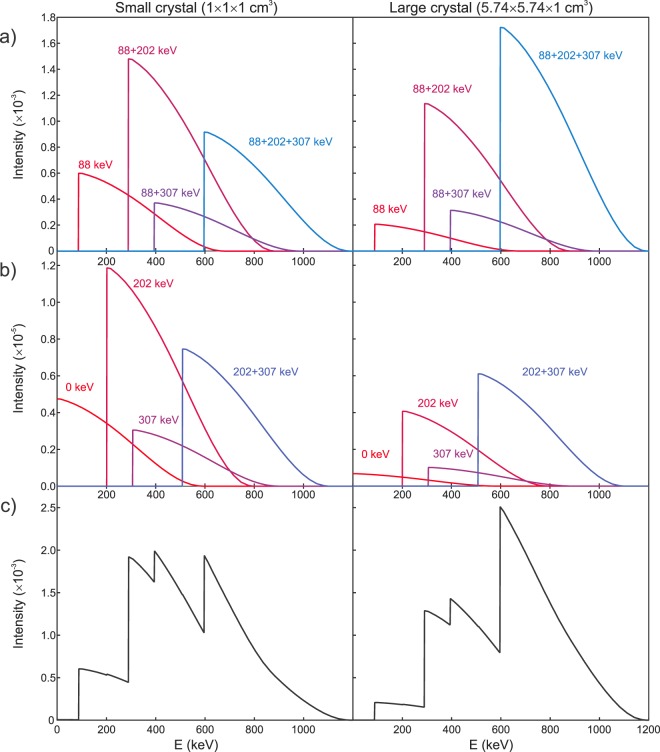
Energy spectra of the different β + γ-ray/internal conversion combinations with (**a**) higher and (**b**) lower probability values. (**c**) Energy spectra obtained from the sum of the individual contributions. Results are shown for two crystal sizes: small cube 1 cm on the left and large 1 cm thick square base prism on the right.

Figure 2a,b show the shifted continuous β-particle energy spectra for each event combination; the area under each curve reflects the probabilities listed in Table 1 for the 88, 202 and 307 keV isomeric transitions of ^176^Hf. The most probable, and thus, the most important peaks are due to the 88 keV, 88 + 202 keV, 88 + 307 keV and 88 + 202 + 307 keV combinations shown in Fig. 2a. In both crystal sizes the probabilities of the spectra in Fig. 2b (0, 202, 307 and 202 + 307 keV) account for less than 1% of the total area. Figure 2c is the sum of the individual contributions shown in Fig. 2a,b to produce the calculated energy spectra (without the Gaussian filtering) for both crystal sizes; the area under each summed spectrum equals unity. Notice that the peak corresponding to all three γ-rays detected in the crystal has a higher intensity for the large prism compared to the small cube because of its larger intrinsic detection efficiency due to the crystal dimensions; this effect is evident in the final energy spectra explaining the differences in the relative peak intensities.

### Experiment vs calculations

**Figure 3:**
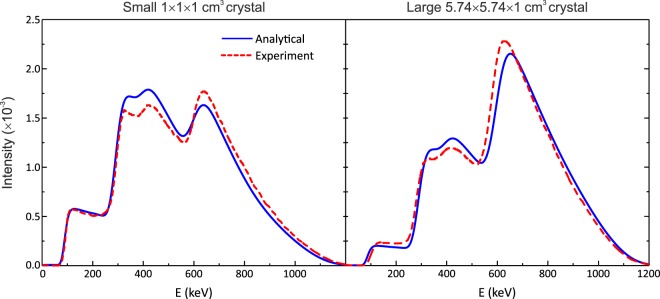
Analytical (solid line), convolved with a varying Gaussian kernel, and experimental (dashed line) LYSO normalized energy spectra.

The calculated (convolved with the varying Gaussian function) and experimental spectra (normalized to have area = 1.0) are shown in Fig. 3. As it can be seen, the calculated energy spectra are able to reproduce the structure observed in both crystal sizes, in good agreement with the experimental data. This agreement is despite of the limitations of this work, namely:

- We have only considered the energy deposited by β-particles, secondary electrons produced from γ-rays interactions and conversion electrons, neglecting the (small) contribution of scattered photons and low energy X-rays escaping the crystal,
- Our calculations do not consider the light transport within LYSO, nor the type of reflector material, which do have an impact through light attenuation in the crystal lattice and light absorption/reflection on the crystal surfaces on the amount of light reaching the photodetector.

In addition, the light coupling material and the proportion of the surface of the crystal in contact with the photodetector can also explain the observed differences. A complete simulation of these processes may help improving the concordance between the experimental and the calculated energy spectra. However, our results demonstrate that the physical mechanisms considered in this work are the most dominant which qualitatively and quantitatively provide an overall explanation of the relative intensities of the observed broad peaks in the background spectrum from LYSO scintillators considering different crystal sizes.

### Extension to different crystal sizes

**Figure 4:**
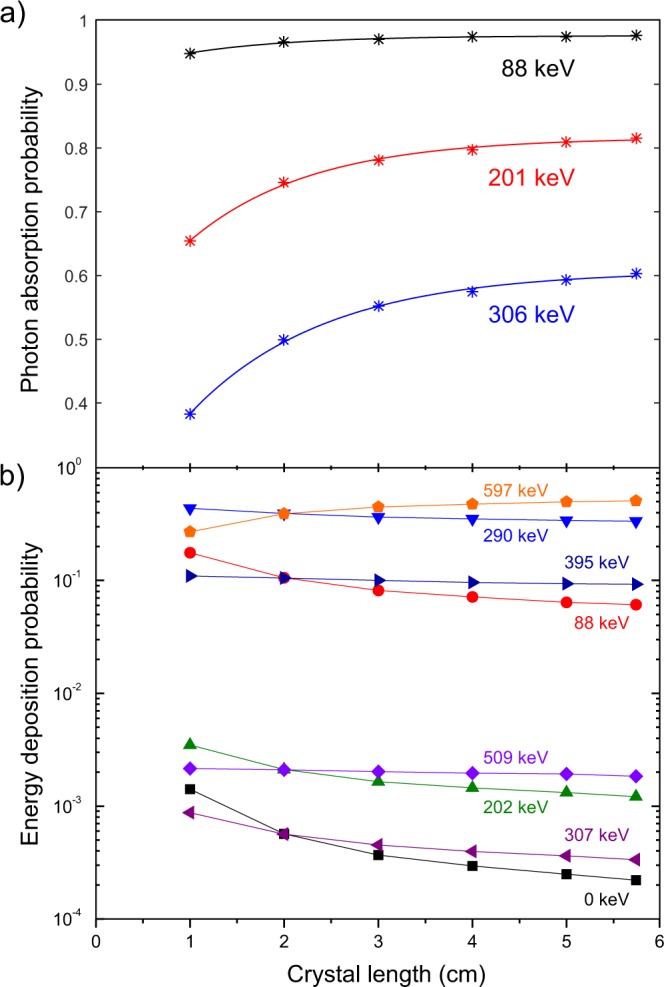
(**a**) Photon absorption probabilities and (**b**) Energy deposition probabilities as a function of crystal square base size. All crystals are square prisms 1.0 cm thick. Values calculated from Monte Carlo simulations with PENELOPE as described in the text.

The Monte Carlo simulations were extended to include the photon absorption probabilities (*P*_*abs*_), equivalent to column 5 in Table 1, of the three gamma rays arising from the isomeric transitions of ^176^Hf for square base LYSO prisms of sides *L* = 2.0, 3.0, 4.0 and 5.0 cm, all 1.0 cm thick. Figure 4a shows these probabilities values (symbols) as a function of *L*. The curves are the best fit of a function of the form:

1 $${P}_{abs}=a(1-b{e}^{{-}\mathrm{cL}}),$$

where *a*, *b* and *c* are parameters obtained using non-linear least-squares fitting (see Table 3). This function resembles the intrinsic efficiency for pencil beam geometry^21^, but clearly this is a very different situation and the coefficients cannot be directly identified with a physical quantity. This fitting, however, provides a practical means to calculate the *P*_*abs*_ for 1.0 cm thick crystals of different square base sizes.

**Table 3:** Best-fit parameters to the data plotted in Fig. 4a.

E (keV)	*a*	*b*	*c*
88	0.9762	0.0666	0.8737
202	0.8165	0.4336	0.7818
307	0.6075	0.7399	0.6956

With these values the energy deposition probabilities were calculated for all crystal square base sizes. A table similar to Table 2 was produced and the results as a function of *L* are shown in Fig. 4b. This figure displays some interesting features. First, it is evident that the different β + γ/IC can be divided into two groups, reflecting the very small probabilities of 0 keV (all 3 γ-rays escaping), 202 keV (only γ_2_ absorbed), 307 keV (only γ_3_ absorbed) and 509 (γ_2_ and γ_3_ absorbed). Also, consider for example the points (circular symbols) for the 88 keV γ_1_-ray: as crystal size increases, the probability of exactly 88 keV being deposited in the crystal reduces with increasing crystal size. This is due to the fact that for larger crystals the probability of self-detection of γ_2_-ray and γ_3_-ray increases, and thus the simultaneous detection of γ_1_ + γ_2_ and γ_1_ + γ_2_ + γ_3_. This translates into a steady decline in relative intensity for low energy peaks and a growth in relative intensity for the high-energy peak.

**Figure 5:**
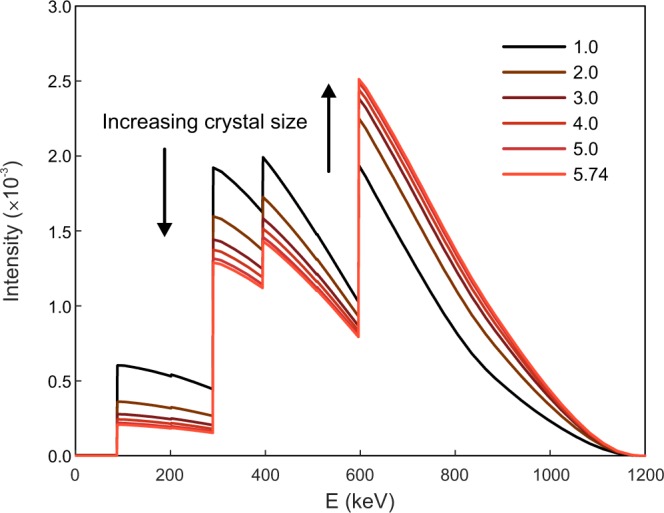
Calculated LYSO energy spectra for 1.0 cm thick square base prisms of different sizes.

Finally, to visualize the evolution of the final energy spectra of LYSO with crystal size, the calculated summed energy spectra for the six square base sizes considered in this work are shown in Fig. 5; recall that all have the same area under the curve equal to 1.0. For the small 1.0 cm cube the three most prominent peaks have a similar intensity, and with increasing crystal square base size, the higher peak energy corresponding to γ_1_ + γ_2_ + γ_3_ increases relative to the others (indicated by the arrows in the figure), following the trends shown in Fig. 4b.

## Conclusions

In this work we have delved into the physical processes responsible for the shape of the energy spectra of LSO/LYSO intrinsic radioactivity in order to gain a deeper understanding of the different contributions of each β-particle and isomeric transition combination. Despite the fact that the scattered radiation and the light transport within the scintillation crystal were not considered in the calculations, our results are in good agreement with the experimental data, explaining the differences observed when using two crystal sizes. Our calculations correctly account for the relative intensities of the peaks observed in different crystals sizes, owing to the detection probabilities for all the β + γ/IC possible combinations.

Our extended calculations for LYSO 1 cm thick square base prisms of different sizes presented in section 3.3 can be extremely useful to predict crystal intrinsic radioactivity as measured by other groups. For instance, the spectra reported by Afanaciev, K. G. *et al*.^12^ for a 10 × 10 × 10 mm^2^ crystal and by Jeong, M. *et al*.^14^ for 50.8 × 50.8 × 10 mm^2^ crystals are in good agreement with our predictions, the latter in spite of using a pixelated crystal array and a different photodetector.

This work may prove very useful to continue using the background signal from the LSO/LYSO crystals to calibrate and assess detector performance (energy linearity response), and may even help in predicting detector energy resolution at different energy values.
